# Supplementary material for: Identification of Freezing-Responsive microRNAs and Their Targets in Chinese Jujube by Small RNA and Degradome Sequencing
Source: Int J Mol Sci. 2026 Sep 4;27(17):7912. doi: 10.3390/ijms27177912 (PMC13566890; doi:10.3390/ijms27177912)
Supplement: Supplementary file 1 [file ijms-27-07912-s001.zip › Supplementary Material S2/novel_miR_229.pdf]

Provisional ID	:	novel_miR_229
Score total	:	0.6
Score for star read(s)	:	-1.3
Score for read counts	:	0
Score for mfe	:	0.3
Score for randfold	:	1.6
Score for cons. seed	:	
Total read count	:	287
Mature read count	:	147
Loop read count	:	0
Star read count	:	140

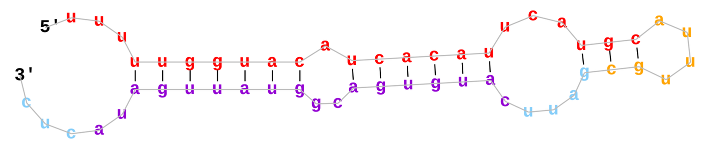

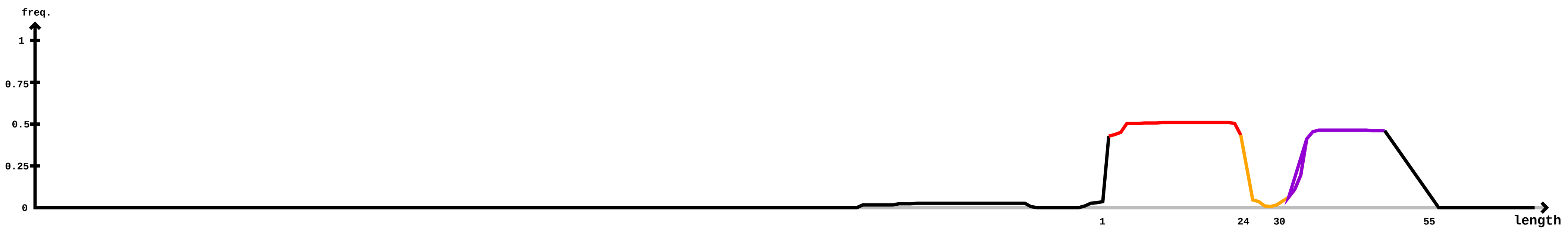


```
                                                                                                                                                                                              Mature                       Star
5'- auguuuucagauacugcuauauaauuacaacccgucuuugcuuaauggauacaaaacacccauacuuacaacggccccugauucaacaagaaauaccaauuuaacaugggcggugguguuuuagugguuguagguggcaagguugcuuuguuaccuauuccauuaggaaccaccgauuuuuugguacaucacauucaugcauuugcgauucaugugacgguauugauacuccuaaaagguguucuauuua  -3'  obs
    auguuuucagauacugcuauauaauuacaacccgucuuugcuuaauggauacaaaacacccauacuuacaacggccccugauucaacaagaaauaccaauuuaacaugggcggugguguuuuagugguuguagguggcaagguugcuuuguuaccuauuccauuaggaaccaccgauuuuuugguacaucacauucaugcauuugcgauucaugugacgguauugauacuccuaaaagguguucuauuua       exp
    .(((.((((......((......................))....)))).))).((((((.............((((.((........................)).))))((((((.((((((((..((((((((((((((...))))))))))))).)))))))))))))))......(((((((.((((((...(((....)))....))))))..)))))))...........)))))).......  reads  mm  sample
    ........................................................................................................................................gcaagguugcuuuguuaccuauuccauu......................................................................................  5  0  JT-1
    ..............................................................................................................................................uugcuuuguuaccuauuccauua.....................................................................................  1  0  JCK-3
    ..............................................................................................................................................uugcuuuguuaccuauuccauua.....................................................................................  1  0  JCK-2
    .................................................................................................................................................cuuuguuaccuauuccauu......................................................................................  1  0  JCK-2
    .............................................................................................................................................................................cgauuuuuugguacaucacauucaug...................................................  3  0  JCK-2
    ..............................................................................................................................................................................gauuuuuugguacaucacauucaug...................................................  4  0  LCK-3
    ..............................................................................................................................................................................gauuuuuugguacaucacauucaugcauuu..............................................  1  0  LT-1
    ...............................................................................................................................................................................auuuuuugguacaucacauucaugc..................................................  1  0  LT-2
    ................................................................................................................................................................................uuuuuugguacaucacauucaug...................................................  1  0  LT-2
    ................................................................................................................................................................................uuuuuugguacaucacauucaug...................................................  1  0  LT-3
    .................................................................................................................................................................................uuuuugguacaucacauucau....................................................  2  0  LT-1
    .................................................................................................................................................................................uuuuugguacaucacauucaugc..................................................  54  0  LCK-2
    .................................................................................................................................................................................uuuuugguacaucaNauucaugc..................................................  1  1  LCK-1
    .................................................................................................................................................................................uuuuugguacaucacauucaugc..................................................  57  0  LCK-1
    .................................................................................................................................................................................uuuuugguacaucacauucaugcau................................................  4  0  LT-2
    .................................................................................................................................................................................uuuuugguacaucacauucaugcauu...............................................  1  0  LT-3
    ..................................................................................................................................................................................uuuugguacaucacauucaug...................................................  1  0  JCK-3
    ..................................................................................................................................................................................uuuugguacaucacauucaug...................................................  1  0  JCK-2
    ..................................................................................................................................................................................uuuugguacaucacauucaugcauu...............................................  1  0  JT-1
    ...................................................................................................................................................................................uuugguacaucacauucauU...................................................  1  1  LT-3
    ...................................................................................................................................................................................uuugguacaucacauucaugc..................................................  1  0  JCK-3
    ...................................................................................................................................................................................uuugguacaucacauucaugc..................................................  1  0  JCK-2
    ...................................................................................................................................................................................uuugguacaucacauucaugca.................................................  1  0  LT-1
    ....................................................................................................................................................................................uugguacaucacauucaug...................................................  7  0  JT-3
    ....................................................................................................................................................................................uugguacaucacauucaug...................................................  2  0  JT-2
    ....................................................................................................................................................................................uugguacaucacauucaugca.................................................  2  0  LCK-3
    ....................................................................................................................................................................................uugguacaucacauucaugcauu...............................................  1  0  LT-1
    ....................................................................................................................................................................................uugguacaucacauucaugcauu...............................................  4  0  LCK-3
    .......................................................................................................................................................................................guacaucacauucaugcauu...............................................  1  0  LT-2
    ..........................................................................................................................................................................................caucacauucaugcauuC..............................................  1  1  LT-1
    ..........................................................................................................................................................................................................uuCcgauucaugugacggu.............................  1  1  LT-2
    .............................................................................................................................................................................................................cgauucaugugacgguauugaua......................  4  0  LCK-3
    ..............................................................................................................................................................................................................gauucaugugacgguauug.........................  2  0  JCK-2
```

Mature **Star**

```
auguuuucagauacugcuauauaauuacaacccgucuuugcuuaauggauacaaaacacccauacuuacaacggccccugauucaacaagaaauaccaauuuaacaugggcgguggugguuuuagugguuguagguggcaagguugcuuuguuaccuauuccauuaggaaccaccgauuuuuugguacaucacauucaugcauuugcgauucaugugacgguauugauacuccuaaaagguguucuauuua
..............................................................................................................................................................................................................gauucaugugacgguauug.........................   1   0   JCK-3
..............................................................................................................................................................................................................gauucaugugacgguauug.........................   1   0   LT-1
..............................................................................................................................................................................................................gauucaugugacgguauugaua......................   1   0   JCK-1
..............................................................................................................................................................................................................gauucaugugacgguauugauacu....................   2   0   LT-2
...............................................................................................................................................................................................................auucaugugacgguauugau.......................   3   0   LT-2
...............................................................................................................................................................................................................auucaugugacgguauugau.......................   1   0   LT-1
...............................................................................................................................................................................................................auucaugugacgguauugauacu....................   1   0   LT-3
...............................................................................................................................................................................................................auucaugugacgguauugauacu....................   2   0   LT-1
................................................................................................................................................................................................................uucaugugacgguauuga........................   3   0   LT-3
................................................................................................................................................................................................................uucaugugacgguauuga........................   2   0   LT-2
................................................................................................................................................................................................................uucaugugacgguauugaua......................   4   0   JT-3
................................................................................................................................................................................................................uucaugugacgguauugaua......................   1   0   JCK-3
................................................................................................................................................................................................................uucaugugacgguauugaua......................   3   0   JT-2
................................................................................................................................................................................................................uucaugugacgguauugauac.....................   1   0   LT-1
.................................................................................................................................................................................................................ucaugugacgguauugau.......................   2   0   LT-3
.................................................................................................................................................................................................................ucaugugacgguauugaua......................   6   0   LCK-3
.................................................................................................................................................................................................................ucaugugacgguauugaua......................   1   0   LT-3
.................................................................................................................................................................................................................ucaugugacgguauugauac.....................   1   0   LT-1
.................................................................................................................................................................................................................ucaugugacgguauugauac.....................   6   0   JT-2
.................................................................................................................................................................................................................ucaugugacgguauugauac.....................   6   0   JT-3
.................................................................................................................................................................................................................ucaugugacgguauugauacu....................   1   0   JCK-2
.................................................................................................................................................................................................................ucaugugacgguauugaCacu....................   2   1   LT-2
.................................................................................................................................................................................................................ucaugugacgguauugauacu....................   1   0   JCK-3
..................................................................................................................................................................................................................caugugacgguauugaua......................   3   0   JCK-3
..................................................................................................................................................................................................................caugugacgguauugaua......................   1   0   JCK-2
..................................................................................................................................................................................................................caugugacgguauugaua......................   29  0   LCK-1
..................................................................................................................................................................................................................caugugacgguauugaua......................   25  0   LCK-2
..................................................................................................................................................................................................................caugugacgguauugauac.....................   5   0   LT-2
..................................................................................................................................................................................................................caugugacgguauugauacu....................   3   0   JCK-2
...................................................................................................................................................................................................................augugacgguauugauacu....................   3   0   LT-3
...................................................................................................................................................................................................................augugacgguauugauacu....................   3   0   LT-1
...................................................................................................................................................................................................................augugacgguauugauacu....................   1   0   JCK-1
...................................................................................................................................................................................................................augugacgguauugauacu....................   6   0   LCK-3
....................................................................................................................................................................................................................ugugacgguauugauacu....................   1   0   LCK-1
....................................................................................................................................................................................................................ugugacgguauugauacu....................   1   0   LT-1
....................................................................................................................................................................................................................ugugacgguauugauacu....................   1   0   LCK-2
```
